# Water clear cell ectopic non-iatrogenic giant parathyroid adenoma in sternohyoid muscle with thyroid nodule and asymptomatic hypercalcemia due to primary hyperparathyroidism: Case report and literature review

**DOI:** 10.1016/j.ijscr.2021.106295

**Published:** 2021-08-10

**Authors:** Walla Mohamed, Walid El Ansari, Mohamed S. Al Hassan, Rayan M. Sibira, Abdelrahman Abusabeib

**Affiliations:** a Department of General Surgery, Hamad General Hospital, Hamad Medical Corporation, Doha, Qatar; b Department of Surgery, Hamad General Hospital, Hamad Medical Corporation, Doha, Qatar; c College of Medicine, Qatar University, Doha, Qatar; d Weill Cornell Medicine, Doha, Qatar; e School of Health and Education, University of Skovde, Skovde, Sweden; f Department of Laboratory Medicine & Pathology, Hamad General Hospital, Doha, Qatar

**Keywords:** Ectopic, Primary hyperparathyroidism, Giant parathyroid adenoma, Water clear cell adenoma, Case report

## Abstract

**Background:**

Ectopic parathyroid gland is not uncommon, and is associated with primary hyperparathyroidism. Giant parathyroid adenoma (PA) and concurrent presence of enlarged thyroid nodule increases the probability of ectopic location. The combination of a giant PA that is ectopic (within the strap muscle) in the neck is very rare, especially in cases with no previous surgery. The rare histopathological findings of the current case, water clear cell parathyroid adenoma (WCCPA), could explain the patient's presentation, since it has low endocrine function.

**Case presentation:**

A 56-year-old Qatari female on routine visit to primary health care physician for hypertension, was incidentally discovered to be hypercalcemic and was referred to the emergency department of our institution. Neck ultrasound showed a thyroid nodule on the left side, but ^99m^Tc-sestamibi scintigraphy identified a left PA. FNAC of the thyroid nodule showed that it was a colloid nodule. She underwent left hemithyroidectomy and excision of left PA. Intraoperatively, the PA was giant and in the sternohyoid muscle. Intraoperative monitoring of intact PTH (IOiPTH) confirmed successful excision.

**Discussion:**

Ectopic giant parathyroid adenoma is rare especially with the intramuscular location in sternohyoid muscle in the neck without previous neck surgeries. The presence of thyroid nodule could be a precipitating factor for migration of the PA. Preoperative assessment with the radiological image is crucial for diagnosis but sometimes fail to localized the PA.

**Conclusion:**

Giant asymptomatic PA with long standing low function before hyperfunctioning should raise the suspicion of WCCPA. If diagnosis is confirmed, metastasis from a clear cell renal cell carcinoma should be ruled out.

## Highlights

• We report a unique rare case of non-iatrogenic ectopic giant water clear cell parathyroid adenoma (WCCPA)
• A parathyroid adenoma >3.5 gm, asymptomatic and with low function before hyperfunctioning, should raise suspicion of WCCPA.
• History, examination, ultrasound, sesta-MIBI scintigraphy and immunohistochemical studies are important for diagnosis.
• High preoperative parathyroid hormone and calcium need monitoring in post-operative period to avoid hypocalcemia.

## 1 Background

Ectopic parathyroid gland refers to a gland located outside of its orthotopic site. This can arise from abnormal migration during embryogenesis or is acquired. Enlarged glands can be displaced from their orthotopic locations due to their size, the influence of gravity, by mass effect from an associated enlarged thyroid; or can be iatrogenic from surgical auto-transplantation [1].

Parathyroid adenoma (PA) is part of a spectrum of parathyroid proliferative disorders that include parathyroid hyperplasia, adenoma, and carcinoma [2]. Ectopic PA (EPA) is not uncommon (3–4% of all PA) [3], and requires a high index of suspicion in both unexplored and re-operative patients [1]. The prevalence of EPA is between 17.5% and 22% in unexplored patients with primary hyperparathyroidism (PHPT) [4], [5], [6]. In re-operated patients, ectopic glands may comprise up to 66% of missed adenomas, making EPAs much more common in the re-operative setting than in the unexplored setting [4], [7], [8], [9], [10], [11].

Most PA are solitary, small in size, and weigh <1 g [2]. Giant PA (GPA) is a rare type of PA weighing >3.5 g [12]. GPAs are rarely encountered, and their surgical management is challenging [13]. It is rare to encounter a PA that is both ectopic and giant, and these characteristics increase the risk of reoperation and morbidity. Most cases in the literature of ectopic GPA (EGPA) are mediastinal, with approximately 20% of parathyroid tumors located in this location [14].

EPA can occur in anatomical locations that may be missed, including thymus (17%), intrathyroidal (10%), undescended glands (8.6%), carotid sheath (3.6%), and retroesophageal space (3.2%) [15]. A GPA that is ectopic in a muscle of the neck (intramuscular) is scarce, and when these are encountered, they are usually not giant. It is extremely rare to find an EGPA in the sternohyoid muscle. Moreover, the combination of such EGPA with an additional thyroid colloid nodule makes it even rarer. Furthermore, PA arising in auto-transplanted tissue can also occur in 4% of cases [16], [17].

In 1994, the first case of a rare variant of PA, the water-clear cell parathyroid adenoma (WCCPA) was described [18]. WCCPA is more common in women than in men, and occur more frequently in the fourth and fifth decades [19]. The severity of hypercalcemia caused by elevated serum parathyroid hormone (PTH) levels determines the symptomatology. Hence, WCCPA has a low endocrine function [20], and clinical signs appear only when it attains a large size and hyperfunctions, resulting in high serum calcium [19].

We report a 56-year-old female with non-iatrogenic ectopic giant WCCPA in the sternohyoid muscle co-occurring with a thyroid colloid nodule and associated with asymptomatic hypercalcemia due to primary hyperparathyroidism. The patient was referred by her family physician to the accident and emergency department of our institution because of hypercalcemia that was incidentally discovered (she was never symptomatic). We report this case in line with the updated consensus-based surgical case report (SCARE) guidelines [21]. In addition, we undertook a literature review of reported cases of WCCPA.

## 2 Case presentation

A 56-year-old Qatari female on a routine visit to her primary health care physician for hypertension was incidentally discovered to have hypercalcemia. The physician referred her to the accident and emergency department at our institution for the management of the asymptomatic hypercalcemia. The patient was seen by the medical team and admitted for investigations and management. Her medical background was significant for hypertension and respiratory compression symptoms, particularly when the patient lied down in lateral position. There was no previous history of neck surgery or irradiation, smoking or alcohol consumption. There was also no family history of thyroid disease or malignancy.

**Fig. 1:**
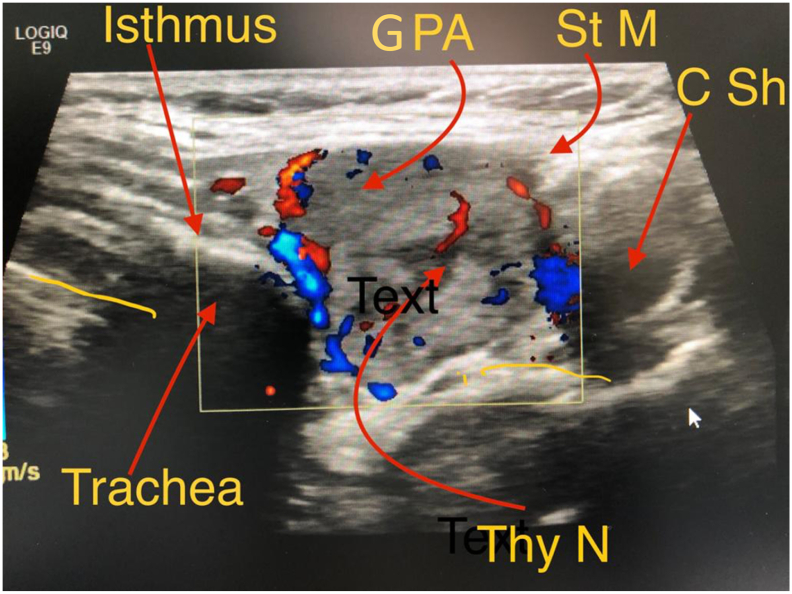
Ultrasound of thyroid nodule and parathyroid adenoma showing well-defined solid mass (21 × 18 × 36 mm) with increased vascularity in relation to the upper pole of the left thyroid lobe and lateral to the isthmus suggesting PA; and heterogeneous solid isoechoic nodule (23 × 16 × 38 mm) in the left thyroid lobe appearing wider than tall, with predominant peripheral vascularity suggesting a left thyroid nodule (GPA: giant parathyroid adenoma; St M: strap muscle; C Sh: carotid sheath; Thy N: thyroid nodule).

**Fig. 2:**
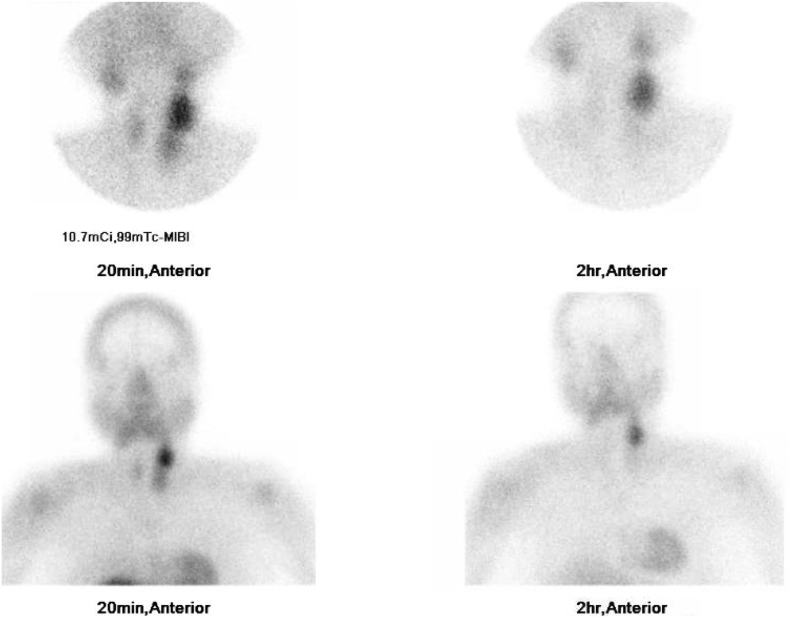
Early and late 99mTc-sestamibi scintigraphy parathyroid scan images of neck and mediastinum anteriorly at 20 min and 2 h showing uptake of radiotracer by thyroid gland along with a large prominent focus at the left superior pole. Delayed images show washout of radiotracer from the thyroid with retained radiotracer activity at the prominent focus. No evidence of ectopic parathyroid tissue in the visualized mediastinum.

Upon clinical examination, the patient had visible and palpable antero-lateral neck swelling (2 × 2 cm) in the upper neck region, not tender, and with no palpable lymph nodes. The systemic review was unremarkable. Her laboratory tests showed high calcium (3.13 mmol/L) and very high parathyroid hormone (PTH 1036 pg/mL) but normal kidney function, suggesting primary hyperparathyroidism. Liver function tests were normal. Ultrasound scan (US) of the neck showed two lesions (Fig. 1) suggesting a left thyroid nodule and left PA. The patient undertook ^99m^Tc-sestamibi scintigraphy, and the findings were consistent with left superior PA (Fig. 2). Ultrasound-guided FNAC of the thyroid nodule revealed a colloid nodule. Hence, diagnosis of left thyroid nodule and PA associated with PHPT was established, and the patient was booked for left hemithyroidectomy with PA excision.

**Fig. 3:**
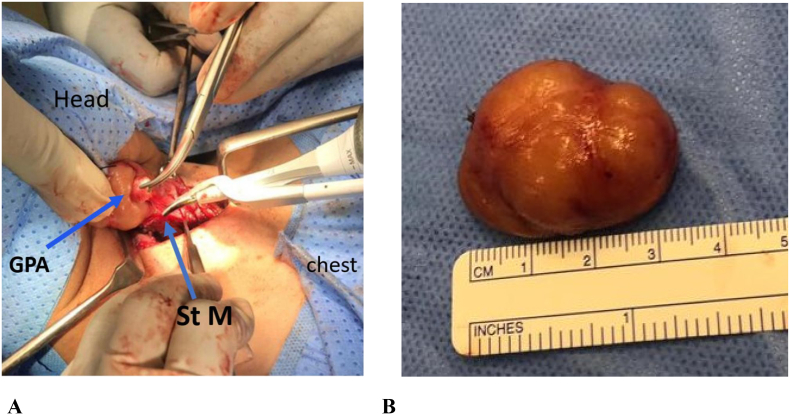
Intraoperative findings: (A) Giant parathyroid adenoma identified intraoperatively (GPA: Giant parathyroid adenoma; St M strap muscle); (B) excised giant parathyroid adenoma.

A senior resident surgeon with assistance and direct supervision of a consultant surgeon performed the procedure. Since the patient had compression symptoms, left hemithyroidectomy with parathyroid adenoma excision were undertaken. Intraoperatively, the left PA was found to be ectopic being intramuscular within the strap muscles, and it was giant (9 gm, Fig. 3). Intraoperative serum level of IOiPTH was done before excision, and after 10 and 20 min of excision, and there was >50% drop in the IOiPTH level. Postoperatively, the patient was kept on calcium gluconate infusion. In the morning of the next day, she received calcitriol 0.5 μg TID, and her calcium level was 2.04 mmol/L. At the time of discharge on the third postoperative day, the patient was doing well, and her serum calcium was 2.20 mmol/L.

Upon follow-up, the patient was seen at our Thyroid Surgery Clinic after a week. She was satisfied with the procedure and had no complaints, normal calcium level, and normal thyroid function. On her second visit to our clinic 3 weeks after her discharge, her thyroid function showed mild hypothyroidism (TSH 21.0 μU/mL, FT4 10.4 μU/mL), and vitamin D level was low (11.0 nmol/L). Hence, the patient was started on thyroxine 50 μg and ergocalciferol 50,000 IU weekly.

**Fig. 4:**
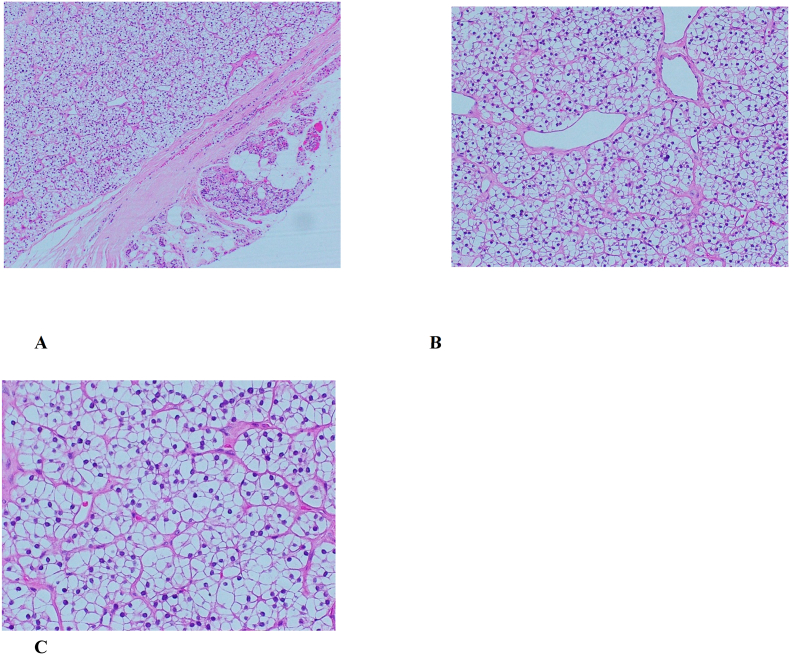
Histopathology of parathyroid adenoma: (A) Water-clear cell parathyroid adenoma. The encapsulated adenoma with adjacent normal parathyroid gland interspaced by adipose tissue (×100); (B, C) higher magnification shows tumor cells composed of monotonous clear vacuolated cells (×200 and ×400, respectively).

Gross appearance of the surgical specimen demonstrated a PA (3 × 2.5 × 1.5 cm, 9 gm) with solid tan homogeneous cut surface. The left thyroid lobe (4.5 × 3.5 × 2 cm) revealed a 1.5 × 1.5 cm well-circumscribed partially cystic solid lesion with focal calcified areas in the upper and middle pole. Microscopically (Fig. 4), there was a benign encapsulated neoplasm composed exclusively of large polyhedral cells with distinct plasma membranes and extensively vacuolated (water-clear) cytoplasm. A rim of normal parathyroid was seen adjacent to the adenoma. A small amount of unremarkable adipose tissue was also present. These findings diagnosed WCCPA.

WCCPA is a rare cause of PHPT. Our literature review showed that only 20 cases have been reported in the literature (Table 1). Histologically, these adenomas are characterized by water clear cells with foamy cytoplasm containing vacuoles [22]. WCCPA must be distinguished from water clear cell parathyroid hyperplasia, in which all four parathyroid glands have water clear cells as their dominant histology [22]. The differential diagnosis of WCCPA includes water-clear cell parathyroid hyperplasia and metastatic carcinoma with clear cell morphology [23]. On other hand, clear cell metastatic carcinomas (specifically clear cell renal cell carcinoma) can have the same morphology and immunohistochemistry of WCCPA [both stain for paired box gene(8) (PAX8), and to some extent to renal cell carcinoma (RCC) monoclonal antibody] [23], [24]. Moreover, RCC can produce PTH-related peptide which results in hypercalcemia, that can also be seen with PA [25]. However, WCCPA, unlike clear cell RCC (CCRCC), stains negative for carbonic anhydrase IX (CAIX).

**Table 1:** Summary of twenty reported cases of waterclear cell parathyroid adenoma.

Case^a^	Age (years)	Sex	Tumor			Calcium	PTH	Symptoms
			Site	Weight (gm)	Size (cm)			
Current case	56	F	SM	9	3	3.13 mmol/L	1036 pg/mL	Asym
Fatih Mehmet (2017) [37]	47	M	L I + R I	0.9 + 1.9	1.6 + 2.5	16.6 mg/dL	744 ng/L	N
Arik (2017) [38]	70	M	Mediastinal	NR	6	NR	NR	Back pain
Pirela (2016) [36]	34	F	L I	NR	2.5	9.3	NR	NR
Chou (2014) [32]	81	F	R S	NR	3.8	NA	450 pmol/L	NR
Tassone (2014) [22]	54	F	L I	NR	2.8	12.4 mg/dL	72 pg/mL	N, GU
Murakami (2014) [35]	59	F	L I	NR	2.8	11.9	72 pmol/L	NR
Piggot (2013) [33]	74	F	L S	13	1.4	12.5	489 pmol/L	NR
Ezzat (2013) [34]	74	F	L I	0.9	1.6	3.13 mmol/L	112 pmol/L	NR
Ezzat (2013) [34]	73	M	L I PA	8	3.7	13	293 pmol/L	NR
Bai (2012) [23]	55	M	R S	0.27	1.4 × 0.8 × 0.6	NR	151 pg/mL	MC, LTH
Papanicolu (2011) [24]	64	M	L I	NR	4.7	Normal	NR	Asym
Kodama (2007) [31]	18	F	R S	21.7	5	11.6 mg/dL	356 pg/mL	N
Kanda (2004) [20]	52	F	L I	15.4	6.8	11.7 mg/dL	672 pg/mL	N
Prasad (2004) [30]	40	F	L superior	4.2	3	12.4 mg/dL	346 pg/mL	F, C, W
Dundar (2001) [28]	43	F	L IThy	NR	6	13.3 mg/dL	1667 pg/mL	Fr, C, F, LTH
Kuhel (2001) [29]	56	F	R S, L S	R 1.7, L 0.5	R 2.8, L 1.5	3.3 mmol/L	52 ng/L	Asym
Beguert (1999) [27]	73	M	L I	NR	2.8	13.8 mg/dL	207 pg/mL	N
Grenko (1995) [26]	40	M	R S	7.6	5	11.3 mg/dL	945 pg/mL	F, C
Kovac (1994) [18]	48	M	NR	NR	NR	11.8 mg/dL	4.5 mIU/mL	N

a Due to space considerations, only the first author is cited; Asym: asymptomatic; C: cramps; F: fatigue; F: female; Fr: fractures; GU: gastric ulcer; I: inferior; IThy: intrathyroidal; L: left; LTH: lethargy; M: male; MC: mood changes; N: nephrolithiasis; NR: not reported; PA: parathyroid adenoma; R: right; S: superior; SM: sternohyoid muscle; W: weakness

## 3 Discussion

We present a rare case of non-iatrogenic EGPA within the strap muscle (sternohyoid) co-occurring with left thyroid colloid nodule and associated with asymptomatic hypercalcemia due to PHPT. In most cases of PHPT due to solitary PA, the adenoma is usually small and weighs <1 gm [2]. Very few cases have been reported where the PA weighed >3.5 gm, referred to as ‘giant’ [12].

In terms of presentation, PA presents classically with PHPT accompanied by recurrent kidney stones, and psychiatric, bone, and gastrointestinal symptoms [12]. However, such full-blown pattern is rarely seen nowadays due to the frequent routine evaluations of patients presenting to the health services [12]. Subsequently, most PHPT cases are being recognized while still asymptomatic [39]. The current patient presented with an antero-lateral neck swelling that she had noted for several years but did not seek medical advice. During a follow-up with her primary care physician for the management of hypertension, her condition was incidentally discovered as she was found to have high calcium level, supporting that the increased use of screening contributes to the early detection of patients [39].

GPA is not necessarily palpable or symptomatic. The weight of the gland correlates with its functionality and thus serum calcium levels [40]. There are only isolated reports of non-functioning GPA [41], [42]. Patients with GPA have higher mean preoperative PTH and serum calcium levels but are less likely to have symptoms of hypercalcemia [43]. The mechanism whereby they are asymptomatic remains unclear [39]. Such asymptomatic patients present later, thus the PA may grow to enormous dimensions before detection [39]. The WCCPA has a low endocrine function [20], hence, clinical signs appear only when the adenoma grows large and hyperfunctions, resulting in high serum calcium [19]. These findings emphasize the importance of detailed history, physical examination, and immunohistochemistry studies in the workup of PHPT. Left parathyroidectomy demonstrated that the parathyroid measured 3 × 2.5 × 1.5 cm, and weighed 9 gm, with solid homogeneous cut surface. In our case, in addition to the clinical findings of the high serum calcium, a visible and palpable mass was obvious in the patient's neck. Cases of palpable PA are extremely rare in the literature [44].

In terms of calcium homeostasis, our patient had high serum calcium and PTH levels. Patients with high preoperative calcium require close postsurgical observation, as feedback effects cause the non-pathologic parathyroid glands to cease their normal function; and resection of the pathologic gland may result in transient hypocalcemia, due to a sudden decrease in calcium level [45], [46]. We agree, as our patient had hypocalcemia in the early postoperative phase that we corrected.

As for investigations, clinical suspicion and laboratory findings followed by imaging studies can confirm the diagnosis. US of the neck is widely used to locate the pathological gland with 75% and 85% sensitivity and specificity respectively [47]. 99mTc-MIBI (sesta-MIBI) scintigraphy has 70%–100% sensitivity [48]. Evidence suggests that mild hypercalcemia, multi-gland disease and co-existing thyroid disease are critical features that influence the sensitivity of preoperative imaging studies, and in such patients, a mini-invasive approach is possible but the use of intraoperative PTH monitoring is required to reduce the risk of unsuccessful surgery [49]. We checked the intraoperative serum level of IOiPTH before excision, and after 10 and 20 min of excision, and there was >50% drop in the IOiPTH level. Preoperative localization of the PA is crucial to achieve an optimal surgical outcome, and the combination of sesta-MIBI scintigraphy and cervical US are considered the best initial way to delineate its anatomic relations [50]. We used these radiological techniques to confirm the diagnosis and localize the PA.

The definitive treatment of primary hyperparathyroidism associated with PA is surgical resection. Preoperative localization of the PA is important, and intraoperative quality control of the surgical excision based on intraoperative serum levels of IOiPTH is a gold standard [51]. In agreement, we undertook IOiPTH that showed a drop to >50% 20 min after excision, indicative of the removal of the PA [48], [52]. Postoperatively, PTH and calcium levels need to be monitored as calcium supplementation is sometimes necessary in order to avoid clinical hypocalcemia [53]. This was the case with our patient and she was started on ergocalciferol 50,000 IU weekly in addition to calcium carbonate 1250 mg 8 hourly per day for two weeks. We followed up the patient at 6 months and one year later and she was symptom-free, serum calcium was within normal limits, and was satisfied with the outcomes of the surgery.

## 4 Conclusion

We report a unique rare case of non-iatrogenic ectopic giant WCCPA. When a PA is giant (>3.5 gm), asymptomatic and with long standing low function before being hyperfunctioning, this should raise the suspicion of WCCPA. If the diagnosis is confirmed, metastasis from a clear cell renal cell carcinoma should be ruled out. Hence detailed history, physical examination, and investigations including ultrasound, sesta-MIBI scintigraphy and immunohistochemical studies are of paramount importance. Complete excision of the adenoma is curative with good outcomes. Where preoperative PTH and calcium are high, monitoring of calcium level in the early post-operative period is imperative to avoid hypocalcemia and its deleterious complications.

## Consent

Written informed consent was obtained from the patient for publication of this case report and accompanying images. A copy of the written consent is available for review by the Editor-in-Chief of this journal on request.

## Provenance and peer review

Not commissioned, externally peer-reviewed.

## Ethical approval

Approved by Medical Research Center, Hamad Medical Corporation reference number (MRC-04-21-068).

## Funding

Nothing to declare.

## Guarantor

Prof Dr. Walid El Ansari: welansari9@gmail.com.

## Research registration number

Not first in Man, hence UIN not required.

## CRediT authorship contribution statement

Walla Mohamed: data collection, data interpretation, writing the paper. Walid El Ansari: study concept, data interpretation, writing the paper. Mohamed S. Al Hassan: study concept, data interpretation, editing the paper. Rayan M Sibira: laboratory and histopathology, data interpretation, editing the paper. Abdelrahman Abusabeib: study concept, data interpretation, editing the paper. All authors read and approved the final version.

## Declaration of competing interest

All authors declare that they have no conflict of interest.
